# Facilitators and barriers to protective eyewear acceptance among Indian farmers: A qualitative study

**DOI:** 10.1186/s12889-025-21655-1

**Published:** 2025-02-05

**Authors:** Gopinath Madheswaran, K Eshwari, Judith Shefali Jathanna, Syed Ibrahim, Krithica Srinivasan, Lakshmi Shinde, Ramesh S Ve, Rashima Asokan, S Saravanan, SB Saranya

**Affiliations:** 1 Acchutha Eye Care & Institute of Optometry, Erode, Tamil Nadu India; 2 Acchutha Eye Care & Postgraduate Institute of Ophthalmology, Erode, Tamil Nadu India; 3 Department of Optometry, Manipal College of Health Professions, Manipal Academy of Higher Education, Manipal, 576 104 Karnataka India; 4 Optometry Confederation of India, Bangalore, Karnataka India; 5 Occupational Optometry Services, Sankara Nethralaya, Medical Research Foundation, Chennai, Tamil Nadu India; 6 Department of Community Medicine, Kasturba Medical College, Manipal Academy of Higher Education, Manipal, Karnataka India

**Keywords:** Farming, Agriculture, Protective wear, Awareness, Qualitative study

## Abstract

**Background:**

Agriculture, one of the most hazardous occupations globally, accounts for a significant proportion of work-related injuries, particularly in developing countries like India. However, lack of awareness and possibly low education levels make farmers in the unorganized sector vulnerable to eye injuries. This study aimed to identify the barriers to and facilitators of protective eyewear among farmers.

**Methods:**

A multicentric study was conducted to assess occupational ocular hazards and preventive strategies among farmers in southern India. The facilitating factors and barriers to spectacles or protective eyewear among the study population were explored using a snapshot qualitative study conducted from two centres in Tamil Nadu and one in Karnataka. Thematic analysis was used to analyse the facilitators of and barriers to protective eyewear among farmers.

**Results:**

Five focus group discussions were conducted with 31 farmers (mean age 55.8 ± 9.2 years) from three centers. Six themes were identified: occupational hazards while farming, practice patterns for managing occupational hazards, uses of protective eyewear while farming, benefits and challenges of protective eyewear, perceptions about protective eyewear and suggestions for improvement. Eye injuries from dust, branches, and chemicals were common, and farmers relied on home remedies for minor issues. Protective eyewear was appreciated for preventing injuries and improving safety but faced challenges such as discomfort, poor fit, and aesthetic concerns, particularly among women.

**Conclusions:**

Although there is awareness regarding the need for protective eyewear, it is often not used due to discomfort during work or concerns about possible breakage of the spectacles. It is imperative for primary eye care professionals to raise awareness regarding the importance of ocular protection in the workplace.

**Supplementary Information:**

The online version contains supplementary material available at 10.1186/s12889-025-21655-1.

## Background

Agriculture is ranked third among the world’s most hazardous occupations, posing risks such as heat-induced disorders, mechanical injuries, insect bites, and toxic effects from chemicals and pesticides [1–5]. In developing countries like India, agriculture is not fully mechanized, but some machinery is used for specific tasks. There is a high prevalence of penetrating eye injuries, ocular surface injuries and corneal ulcerations [6–8]. South India has diverse agricultural practices, including dryland farming, irrigated farming, and plantation. Rice, sugarcane, cotton, tobacco, groundnut, pulses, and vegetables are the main crops, along with tea, coffee, rubber, and spices such as pepper, cardamom, and ginger. Farmers and farm workers are often unaware that they must follow safety guidelines when working with machinery, animals, and chemicals to avoid occupational hazards. They should take breaks to avoid heat stress and wear appropriate protective gear, including eye protection [6–9].

Many farmers in India work in the unorganised sector and may have limited awareness of or access to formal health insurance. However, government initiatives such as the Pradhan Mantri Fasal Bima Yojana and Ayushman Bharat aim to provide coverage to specific segments of the farming population, suggesting that access and awareness may vary across regions and demographic groups [10, 11]. They may have limited education, resulting in a lack of knowledge about maintaining good eye health and safety [12]. Since agriculture is a hazardous profession for eye injuries, it is crucial to understand the risks, educate the community about eye care, and provide corrective/protective eyewear. Eye injuries are a major concern for farmers, affecting 11.3% of those who do not use protective eyewear compared to just 0.7% of those who do. These injuries can seriously impact their livelihoods and quality of life [7, 13–15]. The low uptake of protective eyewear can be attributed to the lack of knowledge and awareness about its importance, economic barriers, cultural beliefs, and concerns about comfort [16, 17].

A survey conducted by Coco Farmers in Ghana identified that the use of ocular protection was reported by 34 participants (6.1%), with the main types being goggles (*n* = 24, 70.6%). Ocular protection was primarily used during chemical application (spraying) (*n* = 31, 91.2%). However, only one participant (0.7%) reported using ocular protection at the time of injury [18]. Measures of eye protection use and of eye safety knowledge and beliefs are based on a survey of 300 Latino farmworkers in North Carolina. Few farmworkers report using eye protection (8.3%); most (92.3%) report that employers do not provide eye protection [19]. In another study by Quandt SA et al., only 7 (8.9%) reported wearing safety goggles or safety glasses at work; the same number reported wearing sunglasses. Only 3 (3.8%) reported wearing face shields for eye protection. Farmworkers’ primary reasons for not wearing eye protection at work were that the device fogged up (35.4%) and was uncomfortable (25.3%) [20]. Globally, few studies have examined occupational-related injuries and awareness of protective eyewear use. However, limited studies have prospectively assessed the importance of protective eyewear with refractive correction in India. The use of protective eyewear and the implementation of comprehensive eye safety programs are crucial for mitigating these hazards and protecting farmers’ vision in the long term. However, there is a need for greater awareness, education, and policy initiatives to address the barriers to the use of protective eyewear among farmers in India. Therefore, understanding the facilitators and barriers to using protective eyewear among farmers in India is crucial in developing targeted interventions, promoting compliance, and preventing eye injuries.

## Methods

A multicentric study was conducted to assess occupational ocular hazards and preventive strategies among farmers in southern India was performed, undertaking institutional research and ethics committee approvals. The study had 2 phases and was conducted totally in 3 centres Sankara Nethralaya, Chennai (SN), Acchutha Eye Care & Institute of Optometry, Erode (AE) and Manipal Academy of Higher Education, Karnataka (MK). Phase 1 of this study involved quantitative research methods, including visual task analysis, comprehensive eye examinations, the prescription of protective eyewear, and referrals for other ocular conditions [21]. In phase 2, facilitating factors and barriers to spectacles or protective eyewear among the study population were explored. The methods and results of phase 2 which are qualitative in nature are presented here as per the Standards for Reporting Qualitative Research guidelines.

### Study design

A snapshot qualitative study-focus group discussions (FGDs) were conducted from two centres in Tamil Nadu and one in Karnataka using a semi-structured interview guide between May 2022 and September 2022. The FGD was carried out based on a thematic descriptive approach. The FGD guide was developed after a literature review and studies exploring facilitators of and barriers to protective eyewear among farmers. A team of occupational optometrists and community outreach practitioners experienced in qualitative research, who also work closely with the farming community, reviewed the FGD guide. The FGD guide had three major domains: (i) ocular hazards in farming, (ii) difficulties faced by the spectacle and/or protective eyewear while farming, and (iii) suggestions to improve protective wear while farming. A copy of the interview guide used to conduct the FGD is provided in supplementary material 1.

### Sampling and recruitment

Participants in phase 1 were contacted, and an FGD was scheduled after two months of spectacle distribution. The inclusion criteria were as follows: only individuals provided with regular spectacle and/or protective eyewear were included for FGD and farmers involved in agriculture (paddy and aracanut) for a minimum of two years and age above twenty years. Farmers with over two years of experience were included to ensure that participants had adequate exposure to farming practices and occupational hazards, enabling them to provide well-informed insights into the use and challenges of protective eyewear. The exclusion criteria were those involved in part-time farming with less than ten hours a week and migration to more than fifteen places in their lifetime. Part-time farmers were excluded from focusing on individuals primarily dependent on agriculture for their livelihood, as they are more likely to encounter continuous and significant occupational risks than those engaged in farming sporadically or as a supplementary activity. Purposive sampling was used to represent age, gender, years of farming experience, and type of work.

### Ethical considerations

Local Institutional Ethics Committee approvals were obtained separately for each centre. Kasturba Medical College and Kasturba Hospital Institutional ethics committee (643/2021) for MK, Institutional Review Board (Ethics Committee)- Vision Research Foundation (1022-2022-P) for SN and Sudha Hospitals- Institutional Ethics Committee- SH/ IEC/ Approval-012/ Jan 2022 for AE. The study protocol was registered in the Clinical Trials Registry of India. The study followed the Declaration of Helsinki. Written consent forms were verbally explained to the participants to ensure that even individuals with limited education could understand the study details before giving their consent. All participants could provide their consent either by signature or thumb impression. Participants were informed about the audio recordings before each FGD started as part of the written informed consent process. The interviewer explained the purpose of the recordings, emphasising their role in accurately capturing the discussion, and reassured participants that their confidentiality would be fully protected. After transcription, the recordings were securely stored in password-protected folders at each center and will be deleted after three years of completion of the study.

### Study setting and data collection

Demographic details like age, gender, years of experience, type of farming and residence in current location were collected from all participants. FGDs were carried out in a commonplace near the residence or workplace of the participant and in the local languages spoken in each centre. A minimum of 6 and maximum 7 farmers participated in each FGD moderated by one of the study investigators at each centre. Both male and female farmers participated in the FGDs, and the moderator ensured that all participants, regardless of gender, had an equal opportunity to express their views. All moderators were formally trained in qualitative research designs as part of their graduate or doctoral research. A total of 5 FGDs were conducted including 31 participants [paddy cultivation (*n* = 19) and aracanut cultivation (*n* = 12)]. No new information was observed following the third FGD (one in each centre). However, two more FGDs were conducted in two centres (each one at SN and MK) to ensure data saturation [22]. All the FGDs were audio recorded; the average duration was 35–45 min, and none of the participants revealed their personal identities during the discussion. During the transcription process, a number was assigned to each respondent to maintain participant anonymity and data confidentiality.

### Data analysis

Descriptive analysis was performed using Statistical Package for Social Sciences V20. The qualitative analysis involved the process of transcription, translation to English, coding and generation of themes. All these were conducted using Microsoft Word 2016. All the individual FGDs were transcribed verbatim by the investigators (GM, JSJ, JS), natives of the study zone and skilled in English and the local languages (Tamil/Kannada). Transcribed content of each FGD was translated to English. Translated content was verified by one of the investigators at each centre. Thematic analysis of the translated content was done to explore the facilitators and barriers to protective eyewear among farmers [23]. A copy of sample thematic analysis is provided in supplementary material 2. The analysis was carried out in the following steps: (1) familiarization with the data, (2) coding the data (deductive and inductive coding), (3) summarizing the codes, developing subthemes & themes, and (4) recoding, reviewing and renaming the themes [24]. The authors (KS, RA) discussed the agreement between the themes and subthemes before confirming the final themes. This process helped achieve internal validity and investigator triangulation [25].

## Results

A total of five FGDs were conducted that included 31 participants from all three centres. In one centre, the main agricultural activity was aracanut cultivation (*n* = 12), while in the other two centres, the focus was on paddy cultivation (*n* = 19). Participant’s mean (± SD) age was 55.8 (9.2) years, years of experience was 33.3 (12.1) years, and residence in the current location was 42.0 (13.9) years. Males were more commonly engaged in farming than females. In contrast, females performed more ploughing, weed removal (deweeding), and cattle maintenance than males. Table 1 shows the demographic details of the participants.

**Table 1:** Demographic details of the participants (*n* = 31)

Demographic details		Number of participants	%
Gender	Male Female	17 14	54.9% 45.1%
Farming type or associated works	Only Farming		
	Male Female	12 3	38.7% 9.7%
	Ploughing		
	Male Female	3 5	9.7% 16.1%
	Weed removal & cattle maintenance		
	Male Female	2 6	6.5% 19.3%

### Facilitators and barriers to protective safety eyewear by farmers

**Fig. 1:**
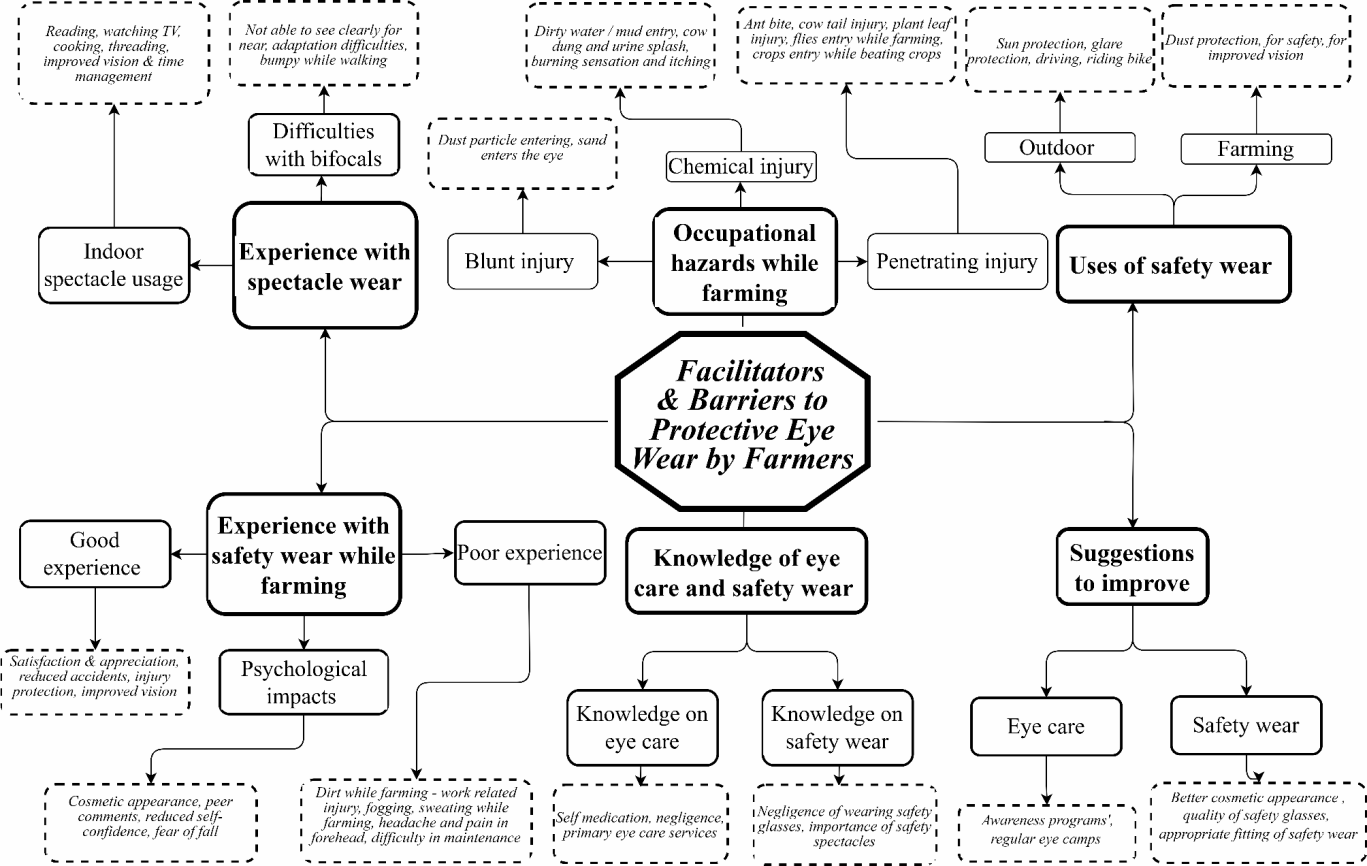
Summary of the themes and subthemes related to the facilitators and barriers participants reported regarding using protective eyewear

Six main themes related to the facilitators of and barriers to protective eyewear by farmers were identified and are presented in Fig. 1. The themes identified were Theme 1: occupational hazards while farming, Theme 2: practice patterns for managing occupational hazards, Theme 3: uses of protective eyewear while farming, Theme 4: benefits and challenges of protective eyewear, Theme 5: perceptions about protective eyewear and Theme 6: suggestions for improvement. The direct quotes from the participants are presented within quotation mark.

### Theme 1: occupational hazards while farming

Participants identified various occupational hazards while farming, including the entry of dust and sand particles during activities such as plucking coconuts or ploughing the land.

*“While plucking a coconut from the tree*,* dust from the coconut tree can fall into the eye…” (FGD 1: MK)*.

*“During harvesting fruits like mangoes and cashews from trees*,* the branches hit suddenly with great force*,* while attempting to pluck fruits from different branches…”(FGD 2: MK)*.

*“While ploughing the land with the machine*,* dust particles*,* and sand can enter the eyes…” (FGD 1: MK)*.

They also reported the risks of cow tail injuries, injuries from vegetative matters (such as tree branches or leaves), and insects entering their eyes while farming.

*“The cow swings its tail at high speed*,* and at that moment*,* dirty water*,* cow dung*,* and urine can fall onto our faces and hands…”(FGD 1: MK)*.

*”While milking the cow*,* the cow’s tail can hit the eyes…” (FGD 1: MK)*.

*”When cutting leaves from trees*,* branches from those trees can hit our eyes and faces…“(FGD 2: SN)*.

*”Insects getting into the eyes is common and happens very often…” (FGD 1:SN)*.

Participants mentioned the potential for chemical injuries while handling fertilizers or coming into contact with dirty water, urine, and cow dung.

*“Farmers*,* while sprinkling fertilizers on the field*,* it may fall on the eyes…” (FGD 1: MK)*.

### Theme 2: practice patterns for managing occupational hazards

Participants mentioned various practices for managing occupational hazards, including self-medication with home remedies such as breast milk or coconut oil for minor eye issues.

*“While threshing*,* grains/husks get into the eyes. We instill breast milk with a force which removes them and aids in quick healing…” (FGD 2: MK)*.

*“I put some coconut oil for cleaning…”(FGD 1: AE)*.

Many also discussed the importance of seeking professional medical care when necessary, mentioning primary eye care awareness and visiting hospitals for treatment.

*“For instance*,* I wash it with water*,* and if it is not cured*,* I visit the hospital…”(FGD 1: AE)*.

*“Yes*,* in PHC itself*,* we get the first treatment*,* and even if it is not cured*,* we go to the government or private hospitals…”(FGD 1: AE)*.

### Theme 3: uses of protective eyewear while farming

Participants mentioned the benefits of wearing protective eyewear during farming tasks.

*“Dust does not fall in the eyes*,* and it protects the eyes from dirt…”(FGD 2:SN)*.

Most mentioned protection against dust and dirt, improved vision, and enhanced safety while performing activities such as planting saplings, harvesting, and preparing fields. Participants appreciated the protective eyewear and acknowledged its usefulness in preventing injuries and improving work efficiency.

*“While preparing the fields*,* transplanting*,* and harvesting*,* the safety eyewear was very useful. It is protective against the grains falling into the eyes while threshing from all sides…” (FGD 1:SN)*.

Few emphasized the safety provided by the protective eyewear, particularly in protecting against dust, pollution, and other environmental hazards.

*“I wear my protective safety glasses when I go to my workplace.” (FGD 2:SN)*.

*“The glasses (safety eyewear) provided are comfortable and soothing to the sun…”(FGD 1:AE)*.

Participants also highlighted other uses of protective eyewear, such as driving during outdoor activities.

*“When driving in a car*,* it is safe; but*,* while riding a two-wheeler*,* often hit by dust and pollution. These glasses (protective eyewear) provide at least 80% protection.” (FGD 1:SN)*.

### Theme 4: benefits and challenges of protective eyewear

Several participants shared the benefits of protective eyewear, citing reduced accidents and injuries from wearing the protective eye wear.

*“No insect will hit*,* no dust will hit*,* no dirt will hit*,* no water will splash…”(FGD 1:AE)*.

*“I had got hit (Cow tail) to the eye but did not cause injury due to these glasses….”(FGD 2:SN)*.

Few expressed satisfaction with improved vision, comfort, and the overall effectiveness of the protective eyewear.

*“If a blind gets vision*,* he will be happy. Likewise*,* the act you gave us the glasses is much appreciated…”(FGD 1:SN)*.

Participants appreciated the protection provided by the glasses and acknowledged their positive impact on their daily activities.

*“Before wearing glasses*,* even if someone is coming at a far distance*,* will not be visible*,* now after wearing visible…”(FGD 1:SN)*.

*“I find both the glasses to be good and comfortable. Wearing at home and working place both are comfortable…”(FGD 2:SN)*.

Few mentioned maintenance difficulties, such as blurry vision when sweating or issues with the fit and grip of the glasses.

*“It will be blurry when it sweats. It comes in between and will become blurry*,* so at that time*,* I will remove*,* wipe and wear…”(FGD 2:SN)*.

Some expressed dissatisfaction with their experiences and found that protective eyewear was less comfortable and convenient for their needs.

*“As it involves more down gaze the glasses*,* they need to be pushed back now and then…”(FGD 2:SN)*.

*“If I wore it for field work*,* dust spots would land on the spectacle and make things less visible…”(FGD 1:SN)*.

### Theme 5: perceptions about protective eyewear

Participants discussed the cosmetic appearance of the protective glasses, noting that they appeared large, especially for females.

*“Now*,* if you see for females*,* it appears a little big…”(FGD 1:SN)*.

Many mentioned peer comments and the importance of self-confidence in wearing glasses. They expressed the need to counter negative perceptions and highlighted the positive impact on their self-esteem and confidence levels.

*“Ah*,* we have said they do not laugh at our glasses because we are wearing it is helpful to us. Dirt will not hit*,* and dust will not hit it is very protective. These are not cataract glasses…”(FGD 1:SN)*.

*“As we age*,* all will have that problem; now you are laughing at us; when you are 40*,* others will laugh at you. What do you know about the glasses you are teasing us? We will say back to them…”(FGD 1:SN)*.

*“No one will ask me*,* and I will say I am wearing it for my vision; my eyesight got reduced*,* so only if I wear these glasses is everything visible to me…”(FGD 1:MK)*.

Very few demonstrated proper knowledge about protective eyewear, understanding of the protective function of the glasses, and the importance of fully covered frames to prevent eye injuries.

*“The frame is fully covered*,* and nothing enters the eye…”(FGD 2:SN)*.

Some participants shared that protective eyeglasses might get damaged or broken while working.

*“While working*,* moving and cutting*,* the instrument might hit the spectacle and break off… so I do not wear it while farming…”(FGD 1:AK)*.

### Theme 6: suggestions to improvement

Participants expressed the need for awareness programs to educate the community about the importance of regular eye check-ups and the availability of appropriate glasses.

*“I did not say we were unaware; we would have come for a check-up and got two glasses; they should have come right…”(FGD 2:MK)*.

*“They were not aware of the advertisement. After seeing us*,* they were worried and expressed that if we had come for these eye screening camps*,* we would have also got these safety glasses…”(FGD 1:SN)*.

Few also emphasized the value of conducting more eye camps and providing free spectacles to support economically low individuals who cannot afford expensive eyewear.

*“People over here are very poor. When they go to a hospital*,* they give them a frame of cost of 3000 INR. That is too much of an expenditure for them. Because of this camp*,* I explained to multiple people about the importance of it (inaudible) and now many people are waiting for these camps… The next time you organize a camp*,* you must help more poor people like us… which will make us happy…”(FGD 2:SN)*.

*“When will be the next camp? Because many other farmers are interested in looking at our spectacles*,* they also want to get the benefit…”(FGD 2:SN)*.

Participants suggested improvements in the cosmesis of protective eyewear, suggesting smaller and more compact designs to address concerns about appearance.

*“I have said that like could have bought a little compact (it could be smaller)…”(FGD 1:SN)*.

Many recommended enhancing the quality and durability of the materials used in protective glasses to withstand the demands of farming tasks.

*“We may need to remove the spectacles on/off*,* mostly in situations like climbing up/down the trees. We may handle the spectacles impatiently those times*,* so we suggest the spectacles should be strong enough for the wear and tear…”(FGD 2:MK)*.

Additionally, participants highlighted the need for improved fitting to prevent glasses from falling off while working and the importance of materials with sufficient strength.

*“During farming*,* if we are looking down*,* it falls. Do something about that… It should not fall off while working…”(FGD 2:SN)*.

## Discussion

The present study explored the experiences and perspectives of participants regarding challenges, benefits, and perceptions of protective eyewear, as well as the practices adopted by farmers to manage potential hazards. The findings shed light on the importance of protective eyewear, the risks farmers face, the effectiveness of protective eyewear, knowledge gaps, and potential areas for intervention.

The entry of dust and sand particles during activities like plucking coconuts or ploughing the land poses a significant risk to their eyes. Injuries caused by cow tails and vegetative matters, such as tree branches or leaves, and the common occurrence of insects entering their eyes while farming highlight the vulnerability of farmers’ eyes to potential harm. Additionally, the exposure to chemicals while handling fertilizers and coming into contact with dirty water, urine, and cow dung further emphasizes the importance of eye protection during farming tasks [6, 8, 20, 26–29]. These findings highlight farmers’ vulnerability to eye injuries and the need for preventive measures, including protective eyewear.

Farmers’ practices for managing occupational hazards revealed a mix of traditional remedies and a growing awareness of the importance of seeking professional medical care. While some farmers relied on home remedies like breast milk or coconut oil for minor eye issues, many recognized the need for professional medical attention when necessary. This indicates the importance of comprehensive education, awareness of primary eye care, and the significance of visiting hospitals for treatment, which is encouraging from a public health perspective [30, 31]. It also signifies the importance of having access to adequate and affordable methods for managing occupational hazards.

Participants highlighted protection against dust and dirt, improved vision, and enhanced safety as the key advantages of using refractive power incorporated protective glasses [15, 32]. The appreciation shown by farmers for the protective eyewear and its role in preventing injuries and improving work efficiency indicates its positive impact on their farming activities. Moreover, the acknowledgment of protective eyewear’s usefulness beyond farming, such as during driving, demonstrates its versatility as a protective measure [29, 32]. Farmers who wear protective eyewear are less likely to experience eye injuries, which can significantly impact their health and productivity. However, the protective eyewear provided in this study consisted only of regular post-operative care spectacles, which did not meet occupational standards or provide refractive correction [14].

The participants strongly expressed the benefits of protective eyewear, with reduced accidents and eye injuries being the most prominent advantage mentioned. The protective eyewear’s improved vision, comfort, and overall effectiveness were also appreciated [33]. However, some participants pointed out maintenance-related challenges, including blurry vision during sweating and issues with the fit and grip of the glasses [34, 35]. These challenges highlight the importance of considering user comfort and practicality while designing protective eyewear. Addressing these challenges would enhance user acceptance and compliance with protective eyewear recommendations [14, 20].

Participants’ discussions about the cosmetic appearance of protective glasses reveal a concern about the size of the eyewear, particularly for female farmers. The fitness-related issues also could be attributed to the fact that the available protective eyewear is not manufactured considering the Indian head and facial anthropometry. Differences in facial features between genders also influence the fit of the protective eyewear, as expressed by a few female farmers, where the frames were larger on their faces and loosely fitting [36]. Few farmers understood the importance of the protective eyewear and were compliant despite negative criticism. The importance of self-confidence in wearing glasses and countering negative perceptions is noteworthy, as it indicates the need for targeted awareness campaigns to address stigma and promote a positive attitude towards wearing protective eyewear. Encouraging self-esteem and confidence among individuals wearing safety glasses can contribute to their acceptance and adoption of protective eyewear. This highlights the importance of considering individual requirements when prescribing protective eyewear and counselling regarding spectacle usage to increase compliance towards protective eyewear usage [14, 32]. Protective eyewear should be designed with safety as the priority, and aesthetics and cosmesis can be considered secondary factors. Compliance can be improved by educating on the benefits of safety eyewear while distributing spectacles.

The suggestions provided by the participants offer valuable insights for improving the accessibility and acceptability of protective eyewear among farmers. Awareness programs and eye camps can be crucial in educating the community about the significance of regular eye check-ups and the benefits of using appropriate protective eyewear. Providing free spectacles to economically disadvantaged individuals can help ensure that eye protection is accessible to all. Furthermore, participants’ recommendations for improvements in the design, cosmesis, and durability of protective eyewear reflect the need for continuous innovation and adaptation to meet farmers’ specific needs and preferences.

Raising awareness through camps and organisations that support sustainable farming practices is essential to promote the use of protective eyewear among farmers. Recommendations should include incorporating refractive correction into protective eyewear to ensure farmers have optimal visual clarity. Currently, the availability of protective eyewear with refractive correction is limited, highlighting the need for customized options that meet occupational safety standards for farmers during their work.

### Strengths

The study included participants with different experiences and agricultural backgrounds, comprehensively understanding the topic. Open-ended interviews allowed participants to share rich and detailed narratives, providing in-depth insights into their experiences and perspectives. The themes that emerged from the data analysis were directly related to the research objectives, highlighting important aspects of occupational hazards, benefits and challenges with safety eyewear, and suggestions for improvement.

### Limitations

The study findings may have been influenced by participant or social desirability bias, as participants might have provided responses they perceived as more socially acceptable. Social desirability bias may have influenced participants’ responses, as they might have felt pressured to provide answers deemed socially acceptable, particularly regarding eye safety practices and health behaviours, given that the same researchers who recommended the use protective eyewear facilitated the FGDs. The findings may be specific to the study participants’ particular region, culture, or farming practices, limiting the generalizability to other settings. The study relied solely on qualitative data, which may limit quantifying the prevalence of specific experiences or perspectives. Participants may have had difficulty recalling specific details or experiences accurately, leading to potential recall bias in their responses.

This study excluded part-time farmers and individuals with less than two years of experience, which may have limited the diversity of perspectives. However, part-time farmers, who often engage in agriculture as a secondary activity, might not reflect true challenges and views about protective eyewear due to their more limited exposure to occupational risks in farming. Additionally, focusing only on more experienced farmers may have overlooked younger or less experienced individuals, who may have different attitudes or awareness of eye protection. This gap highlights the need for further research to explore the experiences of newer entrants into agriculture. Future studies should aim to include a broader range of participants to better inform targeted interventions and address the needs of all farmers.

Despite these limitations, the study provides valuable insights into the experiences, perspectives, and challenges of spectacle wear, occupational hazards, protective eyewear, and eye care knowledge. Future research with more extensive and diverse samples can further enhance our understanding of these factors and help inform interventions and policies to promote eye health and safety.

## Conclusion

In conclusion, this study highlighted comprehensive insights into the perspectives and experiences of farmers regarding the use of protective eyewear and the occupational hazards they face while farming. The identified themes highlight the importance of promoting eye protection in agriculture and addressing protective eyewear challenges and perceptions. By considering these findings, policymakers and public health authorities can develop targeted interventions to enhance eye protection and safety practices among farmers, ultimately leading to improved eye health and well-being in agricultural communities.

## Electronic supplementary material

Below is the link to the electronic supplementary material.

Supplementary Material 1

Supplementary Material 2

## Data Availability

Data can be accessed on request due to privacy/ethical restrictions. The datasets used and/or analyzed during the current study are available with corresponding author on reasonable request. Sample of thematic analysis has been provided in the supplementary file.

## Abbreviations

SN: Sankara Nethralaya, Chennai
AE: Acchutha Eye Care & Institute of Optometry, Erode
MK: Manipal Academy of Higher Education, Karnataka
FGD: Focus group discussion
