# Supplementary material for: Facilitators and barriers to protective eyewear acceptance among Indian farmers: A qualitative study
Source: BMC Public Health. 2025 Feb 5;25:479. doi: 10.1186/s12889-025-21655-1 (PMC11800494; doi:10.1186/s12889-025-21655-1)
Supplement: Supplementary file 1 — Supplementary Material 1 [file 12889_2025_21655_MOESM1_ESM.docx]

**Supplementary Material 1: Interview Guide for Spectacle Compliance**

I request for your consent to record this session and request your good opinion to all our questions and we shall keep these details fully confidential and only abstract summary details will be used to in our presentation with due acknowledgement.

Introductory questions:

1. What is your name?

2. What is your age?

3. Where do you stay?

Transition questions:

4. Since how many years you are working as farmers?

5. Do you do farm in your own land or with others?

6. What type of work you do in farm? (probe: planting sapling, administration of fertilizers etc)

7. For how many years you have done paddy cultivation?

8. For how many years you have done aracanut cultivation?

9. Do you do any other occupation other than farming? (probe like part time job etc)

Specific questions:

10. What are the eye problems you faced while doing farming, before using spectacle or protective

eye wear? (probe: eye infection, dust particle entering eye, pain, redness etc)

11. What did you do when you had those eye problems? (probe: get medicine from pharmacy,

neglect, visit hospital, wash eye etc)

12. Have you started using spectacle/protective eye wear given to you after eye examination?

13. Since how many days you are using it?

14. In a day how long do you use it? (probe: number of hours)

15. When do you wear it? (probe: while doing farming, at home, while going out etc)

16. Do you observe any difference before and after wearing spectacle/ protective eye wear?

17. How did you find spectacle/ protective eye wear useful (benefit)? (probe: while using fertilizer, cleaning crops, from dust etc)

18. Were you able to do all the task wearing spectacle? Any trouble faced while doing some work with spectacle?

19. What are the task you faced problem, while wearing spectacle?

20. What can be done to improve the comfort (while wearing spectacle)?

21. What is the reason for not using the spectacle or for using for lesser hours?

Thank you everyone for participating in this interview
