# Supplementary material for: Facilitators and barriers to protective eyewear acceptance among Indian farmers: A qualitative study
Source: BMC Public Health. 2025 Feb 5;25:479. doi: 10.1186/s12889-025-21655-1 (PMC11800494; doi:10.1186/s12889-025-21655-1)
Supplement: Supplementary file 2 — Supplementary Material 2 [file 12889_2025_21655_MOESM2_ESM.docx]

**Supplementary Material 2- Sample Thematic Analysis**

| **Participant Quotes** | **Codes** |
| --- | --- |
| I am using the spectacle given by you, till now no issues from it, it made things easier because it avoids dust particle entering eye directly while pealing coconut shell. | Farming- Outdoor activities |
| Every day we take out aracanut from tree, coconut from tree and place gobara to all the plants.. this is routine work. This is only the work, other than this no other work. I am using the spectacle given by you, till now no issues from it, it made things easier because it avoids dust particle entering eye directly while pealing coconut shell. That is one and second one is, everything appears clearly through this | Type of Work- Farming- Outdoor activities |
| Every day we take out aracanut from tree, coconut from tree and place gobara to all the plants.. this is routine work. This is only the work, other than this no other work. I am using the spectacle given by you, till now no issues from it, it made things easier because it avoids dust particle entering eye directly while pealing coconut shell. That is one and second one is, everything appears clearly through this | Comfort with glasses= Vision clarity |
| Jasmine plant is there, plucking jasmine flowers, making sides of the field, cows work, taking cow outside. I use spectacle while doing all these works. In this better madam, no problem. | Farming- Outdoor activities |
| One for vision and another for farmers to work. | Safety glasses for farming |
| At first, thank you, on behalf of all farmers. You gave two different types of spectacle. One for vision and another for farmers to work. Farmers while sprinkling fertilizers on the field or while plucking coconut from tree, dust from coconut tree falls. Secondly while plucking aracanut, it falls on the eye, for that one spectacle. | Bifocals for indoors |
| Now the spectacle that we are using are fully covering the eyes, so no problems to the eyes. | Benefits of Safety glasses |
| Now the spectacle that we are using are fully covering the eyes, so no problems to the eyes. | Difficulties without safety glasses |
| Second spectacle means the one used for reading something, or far vision…it helps in seeing. By giving us no problem so far, it is helpful in good way. | Reading- Indoor actitvity- Bifocal glasses |
| we have Jasmine, cow, cutting grass and getting dried leaf, these work I do. Two spectacles were given and one benefit is safety and another worn inside house. So, when one gets dirty the other one can be worn. But one day while tying flower automatically it broke with “tuck” sound and after that i didn’t use. | Benefits of Safety glasses |
| Two spectacles were given and one benefit is safety and another worn inside house. So, when one gets dirty the other one can be worn. But one day while tying flower automatically it broke with “tuck” sound and after that i didn’t use. | Occupational hazards while farming |
| But one day while tying flower automatically it broke with “tuck” sound and after that i didn’t use. | Broken - Poor Safety glasses – Quality of safety glasses |
| Another thing is since it is close here up(WRAPPED SPECTACLE FRAME), morning when i pluck flowers, fog (hanithumbi) covers the spectacle and I have to clean it again and again | Safety while farming |
| Morning when i pluck flowers, fog (hanithumbi) covers the spectacle and I have to clean it again and again. | Fogging -Difficulties with Safety glasses |
| Yes, In that it was fogging, but now in this it is more | Fogging -Difficulties with Safety glasses |
| You will not believe, but I wake up by 4AM and do field work till 11PM. No one works so long. In between i take little rest | Working hours |
| morning after waking up extract cow’s milk, take care of cows and now dairy is little, to see earthworm gobara, to separate waste from nagarasabha and coconut shell comes in larger quantity | Type of Work |
| To avoid spreading of infections, it has to be covered and bifurcation has to be done. We put all these waste at the base of coconut tree and cover it fully | Suggestions to improve Safety glasses |
| For Eight and half to nine hours | Duration of Work |
| while ploughing the land with machine, dust particle and sand enters the eye and while extracting cow’s milk, cow hits with it’s tail to the eye | Farming- Occupational Hazards |
| While sweeping floor dust enters the eyeand while cooking oil splashes to the eye | Indoor Hazards |
| while ploughing the land with machine, dust particle and sand enters the eye and while extracting cow’s milk, cow hits with it’s tail to the eye. While doing mason job, sand particle gets splashed into the eye. While plucking coconut from trees, dust falls. While sweeping floor dust enters the eyeand while cooking oil splashes to the eye. While beating crops and while cutting the crops, it hits the eye and it’s dust enter the eye. | Fogging -Difficulties with Safety glasses |
| No injury, but sand particles used to enter eye and immediately if we wash our eyes it used to go out. | Difficulties while Farming / Occupational Hazards |
| But now with glass dust is avoided. If full black was given then we could avoid sun. dust falling into the eye is avoided | Benefits of Safety glasses |
| If full black was given then we could avoid sun. dust falling into the eye is avoided | Suggestions to improve safety glasses |
| and since far distance vision is given, everything is clearly visible and from very far can be seen. While cutting all worms can be clearly seen. Now we can see. From very far I can see. Small ants was not visible before, but now i can see. | Imporved Vision-Bifocals / Safety glasses- Quality of Life |
| I had got hit to the eye but didn’t cause injury. Dust was falling, small particles were falling, but now it is visible. (VISION IS CLEAR) | Reduced Accidents due to Safety glasses- Quality of Life |
| I was told not yours, first spectacle was given and then exchanged. Far and near vision both are blurred...i can see blur...both are blur | Improper prescription |
| first 5,6 days appeared clear and then blur. Now while watching TV, it appears blur | Adaptation time with Bifocals |
| Protective glass as such i have never used, but for reading rarely i used to use. Now after you increased my power, it is helpful in reading | Knowledge about Safety glasses |
| it is helpful in reading | Reading- Indoor activity – Bifocals |
| Safety for eyes, near is visible. While doing other works i wear. Now everything is visible correctly | Usage of safety glasses |
| Yes, before vision problem started i was wearing. If i wear protective i couldn’t see. While sprinkling oil i used to wear. After vision problem started i stopped wearing protective. Now, in the protective spectacle given by you, no vision related problem. | Improved vision – Safety glasses |
| Yes, because in that (OLD PROTECTIVE SPECTACLE) i could see, but not clear.... everything appeared “white” and nothing fell into the eye, only protection | Problems with Old prescriptions |
| while working if mobile rings, while receiving no problem but to make a call, numbers are not clearly visible | Reading difficulty- |
| Yes, but frame is broken. In the black part (SPECTACLE) there is default. But in fibre (SPECTACLE LENS) there is no problem. In black part only everyone's almost damage | Broken - Poor Safety glasses – Quality of safety glasses |
| : if someone comes they will ask have you undergone eye operation....this is usual...(laughs) | Peer comments- Psychological impacts with safety glasses |
| : if someone comes they will ask have you undergone eye operation....this is usual...(laughs) | Cosmetic appearance- Safety glasses |
| when corona came how we wore mask, just like that...otherwise we don’t wear | Negligence of wearing safety glasses |
| farmers wear and take out spectacle many times, because they have ones climb up one tree and then come down then again climb another tree.....many task involved.....they take out spectacle many times....no patience....so strength should be there and material should be hard | Suggestions to improve quality of safety glasses |
| when will be the next camp? Because many other farmers are interested looking at our spectacles, they also want to get the benefit | Need of Regular camps |

| **Sub Themes** | **Codes** |
| --- | --- |
| Safety wear during outdoor activities | Sun protection |
|  | Glare protection |
|  | Driving |
|  | Riding bike |
| Occupational Hazards while farming | Dust particle entering |
|  | Sand enters the eye |
|  | Cow tail injury |
|  | Ant bite |
|  | Dirty water / mud entry |
|  | Cow dung and Urine Splash |
|  | Flies entry while farming |
|  | Crops entry while Beating crops |
|  | Burning sensation and itching |
| Safety wear while farming related activities | Dust protection |
|  | For Safety |
|  | For Improved Vision |
| Experience with Safety wear | Satisfaction |
|  | Not using safety glasses |
|  | Fear of fall |
|  | Sweating while farming |
|  | Difficulty in maintenance |
|  | Dirt while farming |
|  | Improved Vision |
|  | Dirt- Work related injury |
|  | Importance of Safety spectacles |
|  | Cosmetic appearance |
|  | Peer comments- Psychological impacts with safety glasses |
|  | Reduced Self confidence |
|  | Appreciation |
|  | Fogging |
|  | Reduced Accidents |
|  | Negligence of wearing safety glasses |
|  | Injury protection |
|  | Head ache and pain in forehead |
| Suggestions to improve | Need of awareness programs |
|  | Need of Regular camps |
|  | Better Cosmetic appearance |
|  | Broken - Poor Safety glasses – Quality of safety glasses |
|  | Falling of safety wear |
| Knowledge on eye care | Self medication |
|  | Negligence |
|  | Primary eye care services |
